# Anion Activity and Metastable Phase Formation in Li_1–*x*_FePO_4_ Investigated Using Soft-to-Hard X-ray Absorption and Emission Spectroscopy

**DOI:** 10.1021/acsmaterialslett.4c02389

**Published:** 2025-04-19

**Authors:** Abiram Krishnan, Doyoub Kim, Cherno Jaye, Faisal M Alamgir

**Affiliations:** † School of Materials Science and Engineering, Georgia Institute of Technology, Atlanta, Georgia 30332, United States; ‡ Material Measurement Laboratory, National Institute of Standards and Technology, Gaithersburg, Maryland 20899, United States

## Abstract

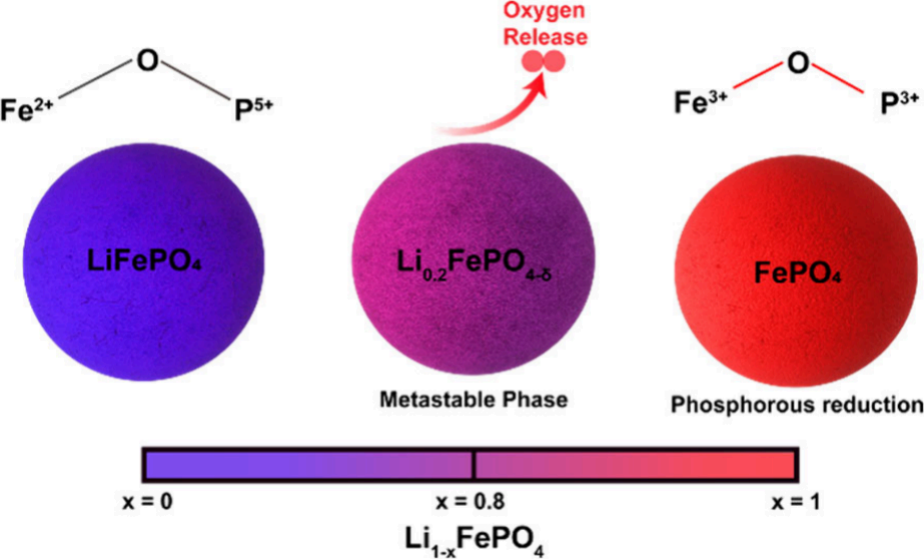

We systematically investigate the intricate roles of cations as well as anions during phase transformation, specifically, the formation of a metastable phase in phospho-olivine, LiFePO_4_ (LFP). The cation- and anion-specific electronic structures are studied using a combination of high-resolution soft-to-hard X-ray absorption and emission spectroscopy. Our findings reveal that the formation of the metastable phase at higher states-of-charge (SoC) is associated with a decreased oxidation state of iron, assisted by oxygen release. Additionally, we find that phosphorus is active in the charge process, exhibiting reduction, resulting from an electron density redistribution between oxygen and its neighboring iron and phosphorus atoms. Furthermore, the phase transformation process in LFP impacts its magnetic properties, with iron retaining its high-spin configuration along with an increased average spin during its transformation into FePO_4_ (FP).

Phospho-olivine based LiFePO_4_ (LFP) was first introduced by Goodenough and co-workers^1^ as a safe and long-cycle-life cathode for rechargeable lithium-ion batteries, that provided an inexpensive, nontoxic, and environmentally friendly alternative to LiMO_2_ cathodes. The stability of LFP cathodes is generally attributed to the strong interactions between the iron 3d and oxygen 2p orbitals. During delithiation, LFP is traditionally understood to undergo a two-phase transformation to FePO_4_ (FP), while retaining its olivine structure as shown in Figure 1a,b. However, both theoretical^2^ and experimental studies^3−6^ suggest the formation of a single metastable phase, isostructural to LFP, during delithiation. It is suggested that the formation of this intermediate phase is significant, bypassing the energy barrier associated with a two-phase process and enhancing the stability and electrochemical performance of LFP as a result. Understanding the mechanisms involved in the formation of the metastable phase and developing techniques to detect it is crucial for improving the cycle stability and efficiency of LFP-based battery systems.

**Figure 1:**
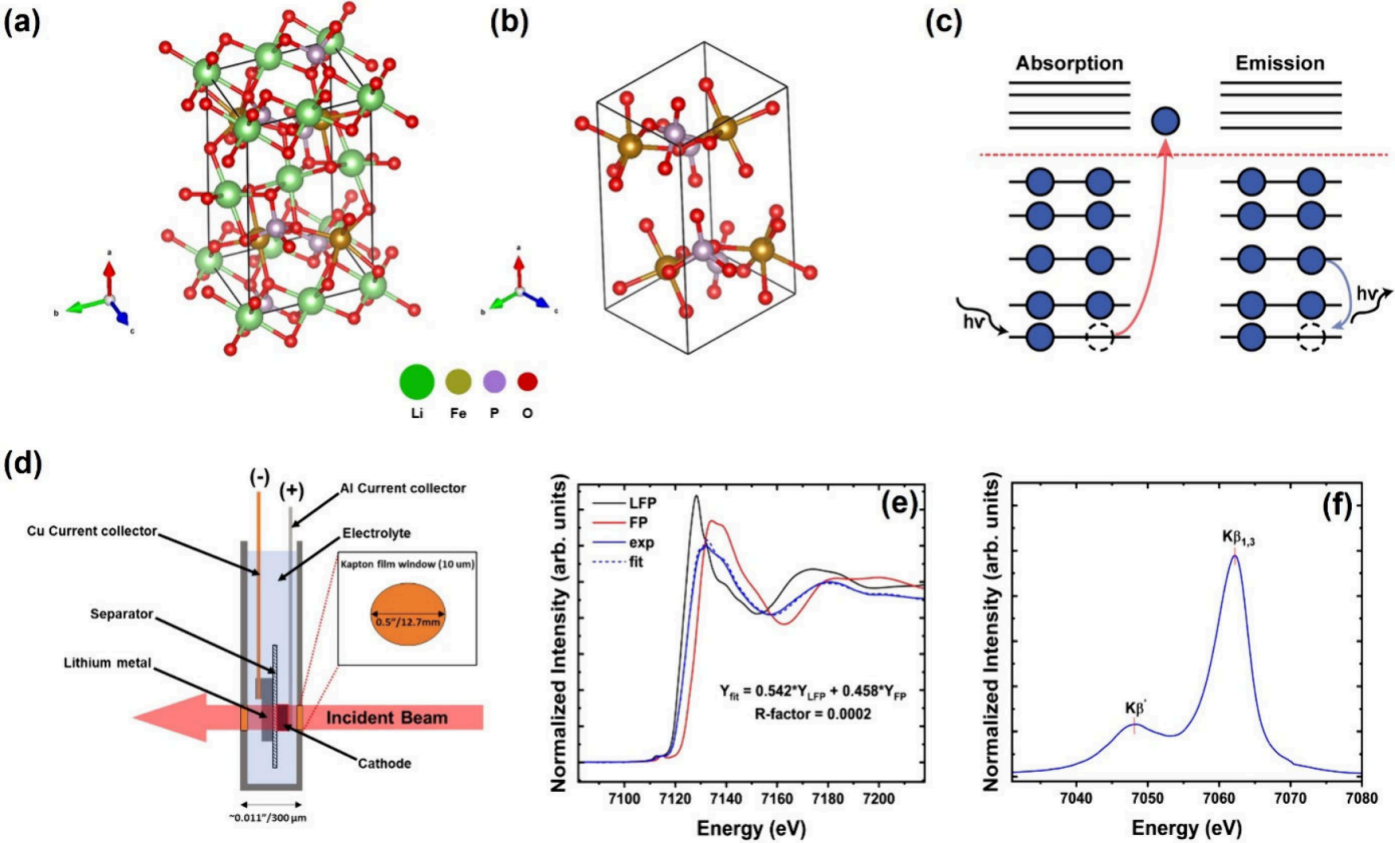
(a) Olivine crystal structure of LiFePO_4_ and (b) FePO_4_ generated using VESTA.^27^ (c) Energy scheme depicting X-ray absorption and emission events. (d) Pouch cell arrangement for operando measurements. (e) Linear combination procedure applied to an unknown measurement of Li_1–*x*_FePO_4_ using LFP and FP as standards. (f) Splitting of iron Kβ emissions (3p-to-1s) for LFP into Kβ and Kβ′ features. The exchange energy difference (Δ*E*_exchange_) is the energy difference between these two features.

The role of oxygen in the redox process of LFP remains unclear, especially its direct and indirect involvement with phosphorus, as there are no current studies that explicate the role of phosphorus. Investigating these processes is challenging due to the need for element-specific probes capable of distinguishing between near-surface and bulk information. The study of metastable phases, which can exhibit a range of structural features including disorder, further complicates this analysis, requiring advanced techniques such as X-ray absorption spectroscopy (XAS) and X-ray emission spectroscopy (XES) to probe the local chemical environment and structure.

XAS and XES, two key core-hole sister techniques capture element-specific local electronic and atomic structure through the generation (XAS) and the quenching (XES) of core-level electron holes.^7,13^ Since Fe, P, and O may each play a role in chemical and structural transformation during (de)lithiation, either XAS or XES can be used, since they probe locally around each of these participating elements. Furthermore, the choice of the specific X-ray energy regimes, i.e. soft, tender, and hard, allows us to manipulate the X-ray source information depth, while the choice of the signal detected (electrons versus X-ray photons) determines the signal’s mean-free path (i.e., the average depth from where the signal emerges). Using combinations of these modes, we can collect information from three different depth ranges from within LFP particles: (1) 10 nm, (2) 100 nm, and (3) 1.5 μm (full particle depth, see Table S1 for more details).

Previous studies using XAS have helped reveal the charge compensation mechanisms in various cathode and anode materials during electrochemical cycling.^8−12^ XAS can be used to examine the impact of phase transformation processes on the electronic structure of surface and bulk elements during (de)lithiation. This capability of XAS is crucial to understand the facile electrochemical removal of lithium from LFP and further elucidate the role of metastable phase formation in improving battery performance. Complementary to XAS, XES probes the electronic and magnetic structure from emitted photons as electrons from higher energy levels quench lower energy core holes created during initial excitation.^13−15^ The energy scheme depicting the absorption and emission events is shown in Figure 1c. The Kβ emission of iron (3p-to-1s) is particularly sensitive to the spin state of the 3d electrons through exchange interactions.^16^ This sensitivity allows XES to capture changes in spin resulting from the phase transformation of LFP during (de)lithiation, thus providing additional insights into the magnetic structure that complement the information obtained from XAS.

In this study, we aim to investigate the mechanisms underlying phase transformation and the role of metastable phase formation in LFP cathodes during charging. High-resolution XAS and XES will be used to systematically investigate the role of transition metals and anions in these processes during delithiation. Depending on the X-ray energy and detection mode, XAS provides detailed, element-specific information about the local atomic and electronic structures, ranging from near-surface to bulk regions. In this work, ex situ samples of Li_1–*x*_FePO_4_ prepared electrochemically at intervals of 10% lithium removal under a slow charge rate of C/50 were used along with operando measurements to analyze changes in the electronic structure around transition metal and anions during the phase transformation from LFP to FP. Furthermore, the changes in spin structure of iron during delithiation will be examined using Kβ XES.

Partial electron yield (PEY) soft X-ray absorption from the Fe L_3_-edge, shown in Figure 2a, is a surface-sensitive technique (≈5 nm information depth) used to investigate the electronic structure of iron through dipole-allowed 2p–3d transitions. The Fe L_3_-edge exhibits two noticeable features at 708 and 710 eV, indicated as (1) and (2) in Figure 2a. A weak isosbestic point is observed at 709 eV, and the lack of a full point of overlap indicates a slight deviation from the ideal two-phase reaction. This deviation is likely due to the formation of the metastable phase on the surface of LFP particles, as suggested by a previous study.^5^ The ratio of intensity of these features, i.e., intensity of feature (2)/(1), shown in Figure 2e, serves as a proxy for the surface oxidation state of iron in Li_1–*x*_FePO_4_.^5^ This ratio increases to its maximum value at *x* = 0.5, suggesting the transformation of LiFe^2+^PO_4_ to Fe^3+^PO_4_ on the surface of the particle. Interestingly, the ratio is found to decrease for 0.6 ≤ *x* ≤ 0.8, indicating a decrease in the oxidation state of iron which could result from the release of oxygen at a higher state-of-charge (SoC). Previous studies have shown that at higher SoC, oxygen is released in the form of CO, which is likely to contribute to this observation.^20^ This trend is also observed through the ratio between intensities of feature (2)/(1) obtained from Fe 2p XPS (Figure 3b) as shown in Figure 2e. The uncertainty in the intensity ratios was found to range between ±2 × 10^–4^ and ±6 × 10^–4^ for sXAS measurements, and between ±3 × 10^–3^ and ±1 × 10^–2^ for XPS measurements. These values were determined based on the background noise levels observed in the respective spectra.

**Figure 2:**
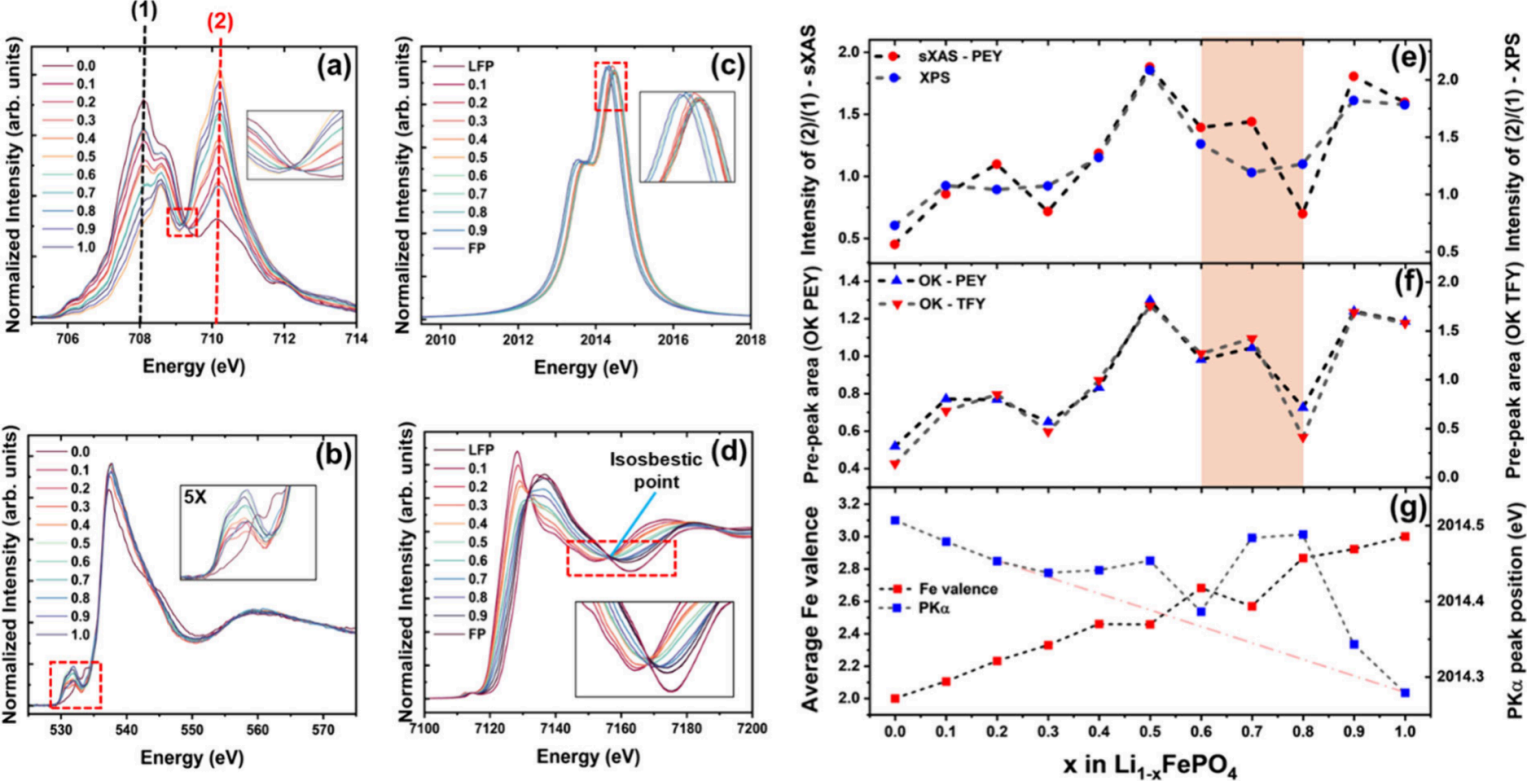
(a) Fe L_3_ measurements collected at PEY for Li_1–*x*_FePO_4_, with the isosbestic point expanded in the inset. (b) O K-edge collected at PEY, with the prepeak feature expanded in the inset. (c) Phosphorus Kα emissions, with the peaks expanded in the inset. (d) Transmission mode Fe K-edge measurements, with isosbestic point expanded in the inset. (e) Ratio of intensities of features (2) to (1) from Fe L_3_ XAS and Fe 2p XPS as a function of lithium removal. (f) O K-edge prepeak area collected at PEY and FY as a function of lithium removal. (g) Bulk-averaged oxidation state of iron obtained from LCA for Fe K-edge, along with phosphorus Kα_1_ peak position as a function of lithium removal. The highlighted region indicates a significant reduction of iron at a higher SoC during the delithiation of LFP.

**Figure 3:**
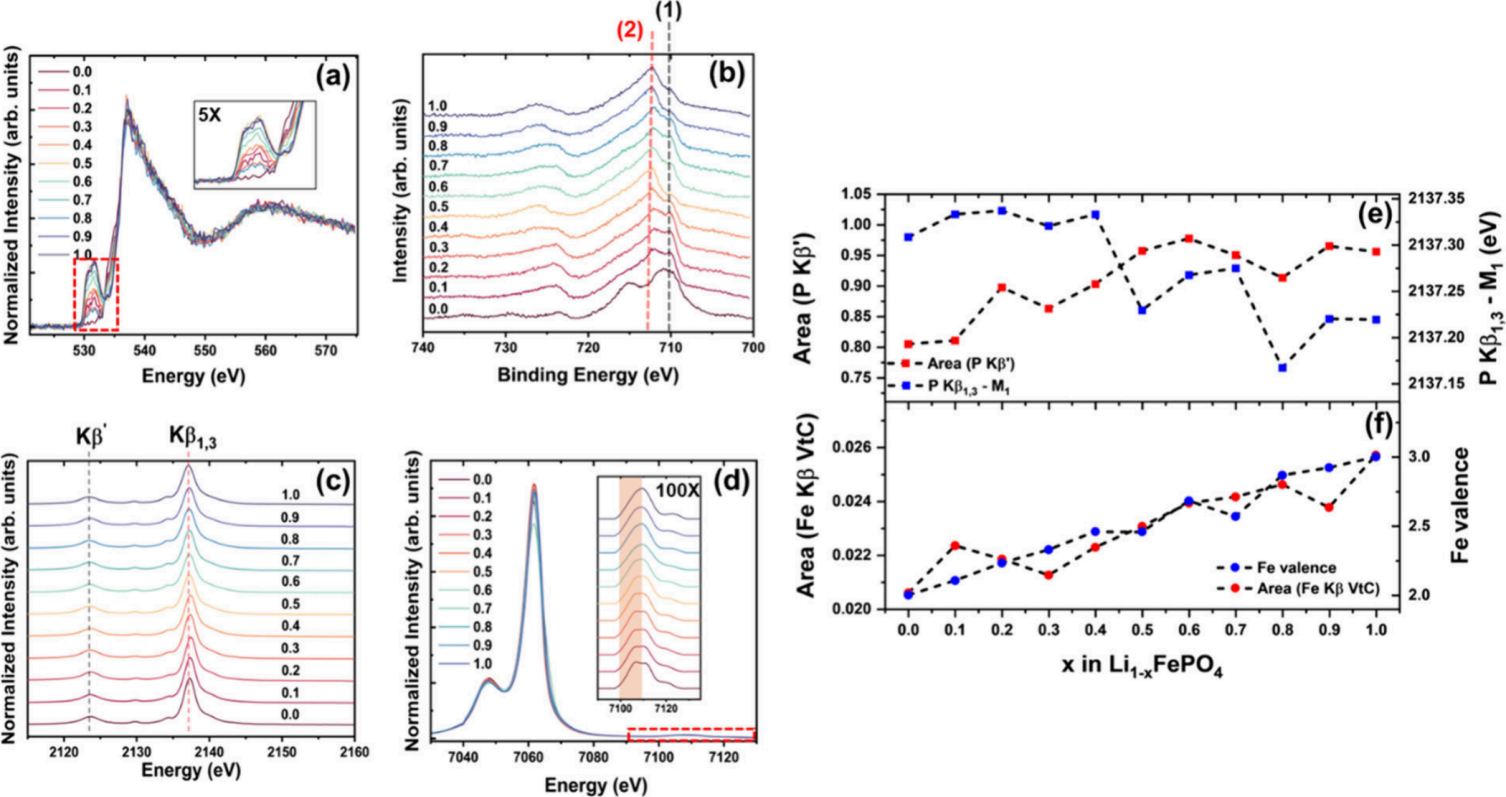
(a) O K-edge measurements collected at FY for Li_1–*x*_FePO_4_ with prepeak feature expanded in the inset. (b) Fe 2p XPS showing the change of surface oxidation state of iron from 2+ to 3+ with delithiation. (c) P Kβ valence-to-core X-ray emission showing the Kβ_1,3_ and Kβ′ features obtained from the hybridization of P 3p and O 2p/2s orbitals. Fe Kβ mainline (3p-to-1s) along with valence-to-core emissions are expanded in the inset. Shaded region highlights shape change of spectra indicating change in Fe–O coordination environment. (e) Area under P Kβ′ feature and the first moments (M_1_) of the P Kβ_1,3_ feature as a function of lithium removal from LFP. (f) Comparison of the area under Fe Kβ valence-to-core emissions and valence obtained from LCA for ex situ samples as a function of delithiation from LFP.

The prepeak feature of oxygen K-edge absorption collected using PEY (inset of Figure 2b), results from the interactions between Fe 3d and O 2p orbitals. The area under this feature serves as a measure of the Fe–O covalency. As shown in Figure 2f, this measure correlates with the oxidation state of iron obtained from iron L_3_ edge absorption, indicating that Fe–O covalency increases with the oxidation state of iron and is notably higher in Fe^3+^PO_4_. When the same feature was measured at fluorescence yield (FY, Figure 3a), which extends the information depth to ≈100 nm,^25^ the area under the prepeak maintains the same trend as PEY (Figure 2f). This indicates that the reduction of iron at 0.6 ≤ *x* ≤ 0.8 is not confined to the surface but extends below the surface of the material. These results further suggest that oxygen vacancies may play a role in reducing iron at higher SoC.

Phosphorus Kα emissions, shown in Figure 2c, correspond to the core-to-core transition from 2p-to-1s orbitals. These emissions exhibit a doublet structure (Kα_1_ and Kα_2_) due to spin–orbit coupling.^21^ The change in the electronic population at the valence shell affects the screening of nuclear charge, resulting in a shift of the Kα lines. The peak position of the Kα_1_ feature as a function of lithium removal (Figure 2g) serves as a proxy for the bulk oxidation state of phosphorus. FePO_4_ is observed to have a lower energy peak position and hence oxidation state of phosphorus compared to LiFePO_4_. Based on literature values of P Kα emissions, where a 0.1 eV shift corresponds to a one-unit change in oxidation state,^23^ the observed 0.2 eV shift between LFP and FP suggests a reduction of phosphorus from 5+ in LFP to 3+ in FP. Additionally, the increase in the area under the phosphorus Kβ′ feature (transitions from molecular orbitals with contributions from the P 3p and O 2s atomic orbitals^22,23^), along with a shift in Kβ_1,3_ peak positions toward lower energies, indicates increased P–O covalency during delithiation (Figure 3c,e). This increase in covalency may result from the generation of oxygen vacancies, which enhance the covalent character between phosphorus and the remaining oxygen atoms.

To investigate the properties of iron within the bulk of the LFP particles, higher energy (hard) X-rays were used to collect Fe K-edge absorption. The near-edge XAS structure shown in Figure 2d exhibits a relatively strong isosbestic point compared to the iron L_3_ measurements due to the relative overrepresentation of bulk information from the hard X-rays. This region can be fit using a linear combination of spectra for LFP and FP standards (procedure outlined in the Experimental Section). The bulk-averaged oxidation state of iron obtained through this procedure is shown in Figure 2g. It is found that the bulk valence of iron increases linearly with minor deviations at higher SoC. This behavior contrasts with the more pronounced variations observed in the Fe L_3_ measurements, suggesting that the effect causing iron reduction near the surface has a limited impact on the bulk of LFP particles under equilibrium conditions.

Iron Kβ_2,5_ emissions shown in Figure 3d (expanded in inset) are valence-to-core transitions reflecting the Fe 3d–O 2p hybrid orbital. These emissions allow us to examine changes in bulk Fe–O covalency and coordination in LFP during delithiation. The iron Kβ_2,5_ spectra for LFP exhibit a doublet structure, typical of high-spin iron complexes with an octahedral coordination environment.^15^ The area under this feature, which measures bulk Fe–O covalency, increases with the oxidation state of iron, as shown in Figure 3f, further confirming the increase in oxidation state of iron with delithiation of LFP particles. Additionally, the feature toward lower energies associated with the doublet structure appears to diminish with lithium removal, changing from a doublet to a singlet structure. This change in shape suggests a decrease in Fe–O coordination^15^ as the oxidation state of iron increases during delithiation.

The phase transformation of LFP to FP can be visualized using a probe that is sensitive to the long-range atomic arrangement, such as X-ray diffraction (XRD). Diffraction measurements of ex situ samples shown in Figure 4a reveal a continuous change in intensities of peaks corresponding to triphylite (LiFePO_4_) and heterosite (FePO_4_) as lithium is removed. While past studies have reported the presence of a metastable phase during continuous charging, evidenced by asymmetric broadening of (200) reflections in XRD spectra toward higher angles,^28^ this is negligible in our ex situ samples under equilibrium conditions. This observation is further supported by the strong isosbestic point of the near edge structure (Figure 2d) and the low *R*-factors obtained from LCA using LFP and FP as the standard, as shown in Figure 4f. The phase fraction of FP with lithium removal, determined by using Rietveld refinements with LFP and FP as the two phases, closely matches the predictions from LCA of the near-edge structure (Figure 4e). This indicates a synchronous change in the electronic and local environment as LFP transforms into FP under relaxed/near-equilibrium conditions in ex situ samples.

**Figure 4:**
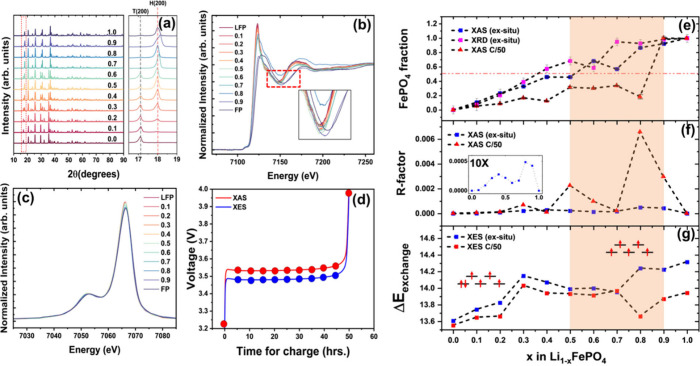
(a) Diffraction patterns for Li_1–*x*_FePO_4_ with the (200) reflection expanded. (b) Fe K-edge spectra collected under operando cycling at a slow C-rate of C/50, with the weak point shown in the inset. (c) Fe Kβ emissions collected under operando cycling at a C-rate of C/50 (ex situ Fe Kβ emissions shown in Figure 3d). (d) Voltage profile for operando XAS/XES measurements, including the times at which spectra were collected during delithiation. (e) FP fraction as a function of delithiation, predicted by ex situ XRD and XAS measurements and compared with operando XAS measurement. (f) *R*-factor, a measure of deviation from the two-phase model, calculated for both ex situ and operando measurements. (g) Exchange energies as a function of delithiation for ex situ and operando measurements, with a schematic showing the electronic configuration of the 3d shell for LFP (left) and FP (right). The highlighted region indicates a correlation between the formation of the metastable phase and reduced bulk oxidation and the spin state of iron at a higher SoC during the delithiation of LFP.

Unlike the ex situ measurements, which measure equilibrated structure, operando experiments using XAS are critical in capturing kinetic-limited phenomena during battery operation.^24^ The near-edge structure of the Fe K-edge collected under a small C-rate of C/50 is shown in Figure 4b. The spectra exhibit a weak isosbestic point at around 7150 eV, with significant deviations from the two-phase field occurring at a higher SoC. LCA using LFP and FP as the standards yields the FP fraction as a function of lithium removal, as shown in Figure 4e. Under operando conditions, the FP content shows significant lag from ex situ measurements, with the FP fraction exceeding 50% at *x* ≥ 0.8. This lag in material response to electrochemical changes could be attributed to the low electronic and ionic conductivity of LFP.^25^ Surprisingly, despite using 10% conductive carbon and operating under slow rate of C/50, this effect remains pronounced. This lag in iron’s response to lithium removal points to a temporary charge compensation mechanism that involves the anions. To further understand the unusual behavior observed in operando experiments, the LCA *R*-factor, which is used to identify deviations from the two-phase field and hence the amount of metastable phase, was calculated according to eq 1 (Figure 4f). The *R*-factor was significantly higher in operando measurements for 0.5≤ *x* ≤ 0.8, which correlates with regions of reduced oxidation state of iron, suggesting the formation of an oxygen-deficient metastable phase of the form Li_1–*x*_Fe^2+^P^(5+*x*–2δ)+^O_4−δ_, where δ is the amount of oxygen removed from LFP. In contrast, ex situ measurements show approximately ten times lower deviations in this range, indicating the limited and transient nature of this nonequilibrium phase. Scheme 1 proposes a potential mechanism summarizing the phase transformation process, which involves the formation of a metastable phase that charge compensates not through iron but via oxygen vacancies. This intermediate phase relaxes into the final structure, where iron is oxidized to 3+ and phosphorus is reduced to 3+, with the loss of one oxygen atom.

**Scheme 1:**
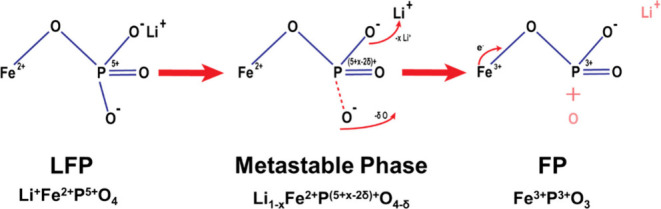
Potential Phase Transformation Mechanism Where LFP Transitions to FP via a Metastable Intermediate Phase, with Charge Compensation Occurring through the Loss of Oxygen

To understand the effect of the phase transition on magnetic properties, iron Kβ emissions were obtained, as shown in Figure 4c. These emissions are sensitive to the spin of the 3d orbital of iron through 3p–3d exchange interactions.^13^ The strong presence of the shoulder feature at ≈7052 eV indicates that iron retains its high-spin state^29^ with delithiation, consistent with past XAS studies.^26^ The exchange energy difference (Δ*E*_exchange_), obtained from experimental spectra, serves as a proxy for changes in the spin state of the 3d shell. This value was obtained for samples under ex situ (Figure 3d) and operando conditions (Figure 4c) as shown in Figure 4g. Both measurements show increased values of Δ*E*_exchange_, and hence spin, with lithium removal from LFP. A sharp decrease in spin is observed at *x* = 0.8 for the operando samples, further confirming the reduced oxidation state of iron due to the formation of nonequilibrium phase via generation of oxygen vacancies, resulting in a lower spin state. The voltage profiles for operando XAS and XES measurements, along with the times at which spectra were acquired, are shown in Figure 4d.

In summary, the mechanisms behind the formation of a metastable phase in LFP cathodes during delithiation were investigated using soft-to-hard XAS and XES. Our findings reveal that phosphorus undergoes reduction during delithiation due to the redistribution of electron density around oxygen atoms shared between iron and phosphorus in LFP. Deviations from the two-phase transformation model at high SoC, along with a reduced oxidation state of iron observed both in bulk and near the surface, suggest that oxygen release facilitates the formation of a metastable phase of the form Li_1–*x*_Fe^2+^P^(5+*x*–2δ)+^O_4−δ_. Charge compensation during the formation of this phase occurs through the generation of oxygen vacancies, allowing iron to retain a 2+ state while also leading to the reduction of phosphorus. Furthermore, our examination of the iron spin structure using Kβ XES indicates that iron retains its high-spin configuration and shows an increase in the average spin during delithiation. These results underscore the intricate role of anions in the phase transformation process of LFP cathodes in lithium-ion batteries.

## Experimental Section

### Cell Assembly and Electrochemical Cycling Protocol

The cathodes used in this study were prepared by using the slurry casting method. The active material, LFP with an average particle size of 1.5 μm purchased from MSE Supplies, was mixed with conductive carbon black and poly(vinylidene difluoride) (PVDF) using a mass ratio of 8:1:1. *N*-Methyl-2-pyrrolidine (NMP) was used as the solvent to create a homogeneous slurry. The slurry was coated on an aluminum current collector using a film applicator with a gap setting of 250 μm and dried for 24 h in a vacuum oven at 100 °C (SEM image of the electrode is shown in Figure S1). The lithium half-cells were assembled inside an argon-filled glovebox to prevent contamination, using coin cells for ex situ samples and a pouch cell setup for conducting operando measurements (Figure 1d). A polypropylene membrane (Celgard 2500) served as the separator, and the electrolyte was composed of 1 mol/L LiPF_6_ in a 1:1 volume ratio of ethylene carbonate (EC) to diethyl carbonate (DEC). The cells were cycled under a constant current corresponding to a slow C-rate of C/50, meaning the cells were fully charged over a period of 50 h. This slow rate was chosen to reach deeper charge levels and enable detailed phase transformation analysis for both ex situ and operando measurements.

### XAS and XES Measurements

XAS measurements of Fe K-edge along with XES measurements of P Kα, P Kβ, and Fe Kβ were carried out using a laboratory-scale instrument that employed the Rowland circle geometry to achieve energy tunability of X-rays generated by a bremsstrahlung source.^17^ The setup used for operando XAS/XES measurements is shown in Figure S2. Soft XAS measurements of Fe L_3_ and O K-edges were collected using beamline 7-ID-1 at NSLS-II at Brookhaven National Laboratory. XAS data were normalized and processed using Athena^18^ from the Demeter software package. XES measurements were normalized based on the area under the spectra before analyzing the intensity, area, and peak positions.

### Linear Combination Analysis Using Fe K-Edge to Detect Metastable Phase

Linear combination analysis (LCA) for Fe K-edge was performed using Athena^18^ which is part of the Demeter software package. The experimental spectra for Li_1–*x*_FePO_4_ were fit by using a linear combination of spectra obtained for LFP and FP standards, as illustrated in Figure 1e. FP standard was prepared by chemical oxidation of LFP using NO_2_BF_4_ in acetonitrile solvent followed by rinsing and vacuum drying at 100 ^o^ C. Deviations from the expected two-phase transformation during lithium removal, which are indicative of the metastable phase, are measured using the *R*-factor, defined as
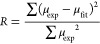
1where μ_exp_ and μ_fit_ are the absorption coefficients of the experimental and fit spectra.

### Sensitivity of XES Kβ Emissions to Spin via Exchange Interactions

The chemical sensitivity of XES Kβ emissions (3p-to-1s) for 3d transition metals arises from interactions between electrons in 3p and 3d orbitals.^13^ These interactions cause the emission line to split into the Kβ_1,3_ mainline and the Kβ′ shoulder feature, as illustrated in Figure 1f. The energy difference between these features,^16^ resulting from parallel (Kβ_1,3_) and antiparallel (Kβ′) alignment of 3p and 3d electrons, is influenced by the spin of the valence d shell and is given by
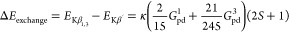
2where κ is the scaling factor, *G*_pd_^1^ and *G*_pd_^3^ are Slater–Condon parameters,^19^ and *S* is the spin of the valence d shell. An example of the sensitivity of these features to 3d spin changes in iron compounds is shown in Figure S3. The energy difference is sensitive to spin, with the extent of the change in the energy difference controlled by the scaling factor and Slater–Condon parameters according to eq 2.

### X-ray Diffraction Measurements and Rietveld Refinement for Phase Fraction Estimation

X-ray diffraction patterns for ex situ samples were collected between 10° and 90° at room temperature using Cu Kα radiation with a wavelength of 1.54 Å. Rietveld refinement was performed using Rigaku SmartLab Studio II software to estimate the phase fractions. LFP (PDF# 65-0257) and FP (PDF# 65-0258) were selected as the reference phases. The refinement details for ex situ measurements are provided in Table S2. The reliability parameters (*R*_wp_) obtained for all fits used in this study, shown in Figure S4, are below 5%.

### X-ray Photoelectron Spectroscopy (XPS)

Fe 2p core level photoemission spectra for LFP cathodes were collected using a monochromatic Al Kα source (1486.6 eV) under ultrahigh vacuum conditions. The binding energy scale was calibrated using the C 1s peak at 284.8 eV as a reference.

## Supporting Information Available

SEM image of LFP electrodes; instrument setup for operando XAS and XES measurements; Kβ XES plots for different iron compounds; information depths collected using these techniques; Rietveld refinement details for ex situ XRD (PDF)

## Abbreviations

XAS: X-ray absorption spectroscopy
XES: X-ray emission spectroscopy
LFP: LiFePO_4_
FP: FePO_4_
XRD: X-ray diffraction
XPS: X-ray photoelectron spectroscopy
LCA: linear combination analysis
SoC: states-of-charge
