# Supplementary material for: Anion Activity and Metastable Phase Formation in Li1–xFePO4 Investigated Using Soft-to-Hard X-ray Absorption and Emission Spectroscopy
Source: ACS Mater Lett. 2025 Apr 19;7(5):1956–62. doi: 10.1021/acsmaterialslett.4c02389 (PMC12056757; doi:10.1021/acsmaterialslett.4c02389)
Supplement: Supplementary file 1 — tz4c02389_si_001.pdf [file tz4c02389_si_001.pdf]

Supporting Information for:

Anion Activity and Metastable Phase formation in $Li_{1-x}FePO_4$ Investigated Using Soft-to-Hard X-ray Absorption and Emission Spectroscopy

Abiram Krishnan[1], Doyoub Kim[1], Cherno Jaye[2], and Faisal M Alamgir[1]*

[1] School of Materials Science and Engineering, Georgia Institute of Technology, Atlanta, Georgia 30332, United States
[2] Material Measurement Laboratory, National Institute of Standards and Technology, Gaithersburg, Maryland 20899, United States

Corresponding Author
*faisal.alamgir@mse.gatech.edu

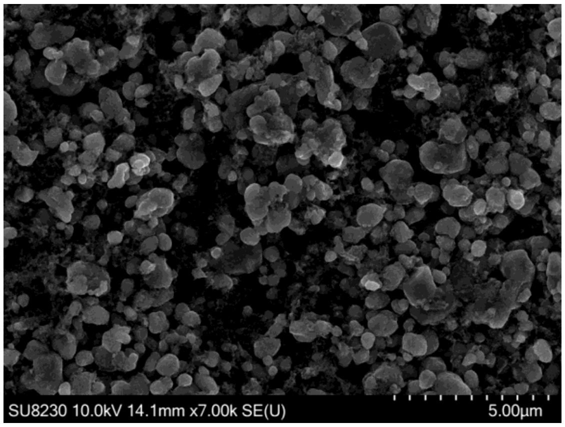

Figure S1: SEM image of $LiFePO_4$ electrodes with carbon additive and binder. The average particle size of $LiFePO_4$ particles used in this study is 1.5 µm.

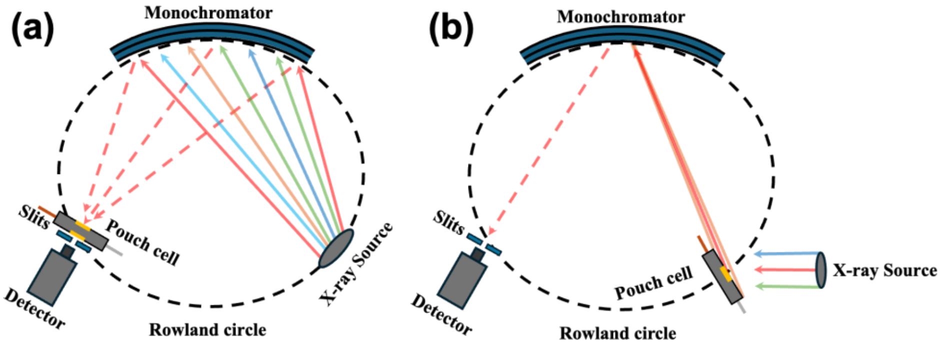

Figure S2: Operando setup for (a) XAS and (b) XES measurements carried out using a laboratory-scale spectrometer.

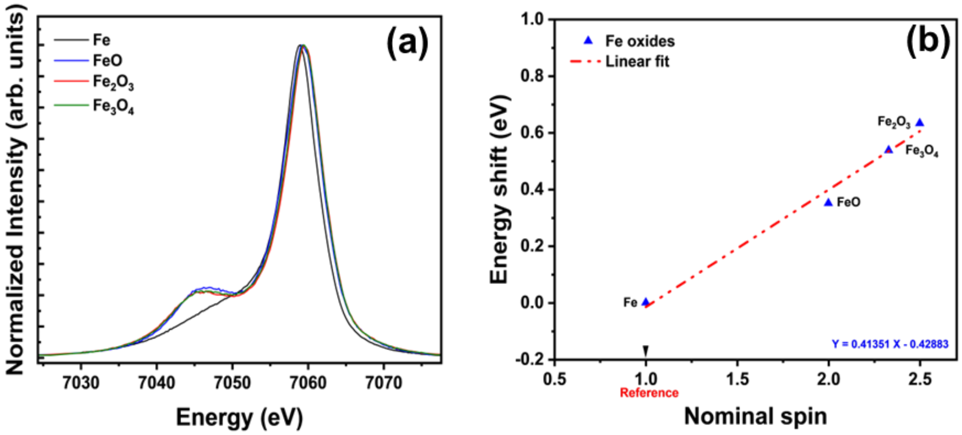

Figure S3: (a) $K\beta_{1,3}$ x-ray emission spectra for iron oxides along with a plot of (b) energy shift relative to iron metal reference against nominal spin. There exists a linear relationship between the energy position of the peaks and the nominal spin of the iron compounds.

Table S1: Information depth obtained from LFP particle of average particle size of 1.5 µm using different element-specific probes with different energies and collection modes. In the table, "s", "t", and "h" refer to soft, tender and hard x-rays, respectively.

Element-specific probe	Information depth
sXAS – Fe L_3 and O K-edge	PEY: ≈5 nm FY: ≈100 nm
hXAS – Fe K-edge (transmission mode)	≈1.5 µm (full particle depth)
hXES – Fe Kβ	≈1.5 µm (full particle depth)
tXES – P Kα and Kβ	≈1.5 µm (full particle depth)
XPS – Fe 2p	≈10 nm

Table S2: Rietveld refinement details for ex-situ $Li_{1-x}FePO_4$ samples assuming LFP and FP as the two phases.

X in $Li_{1-x}FePO_4$	Triphylite (LFP)							Heterosite (FP)							$\%R_{wp}$
	a(Å)	b(Å)	c(Å)	α°	β°	γ°	wt%	a(Å)	b(Å)	c(Å)	α°	β°	γ°	wt%	
0.0	10.32271	6.00392	4.68966	90	90	90	99.80	9.33	6.57	4.998	90	90	90	0.20	1.98
0.1	10.32018	6.00300	4.69146	90	90	90	92.36	9.8187	5.7939	4.7821	90	90	90	7.64	2.58
0.2	10.32099	6.00318	4.69084	90	90	90	79.42	9.8194	5.7930	4.7833	90	90	90	20.58	2.60
0.3	10.3207	6.00285	4.69068	90	90	90	60.96	9.8152	5.78946	4.78118	90	90	90	39.04	2.22
0.4	10.3186	6.00209	4.69212	90	90	90	42.63	9.8150	5.78959	4.78072	90	90	90	57.37	3.45
0.5	10.31901	6.00179	4.68969	90	90	90	31.73	9.81276	5.78815	4.78082	90	90	90	68.27	2.55
0.6	10.3203	6.00254	4.69057	90	90	90	48.65	9.8139	5.78857	4.78137	90	90	90	51.35	2.33
0.7	10.326	5.8838	4.731	90	90	90	5.00	9.8137	5.78823	4.78160	90	90	90	95.00	2.39
0.8	10.3205	5.9996	4.6933	90	90	90	7.30	9.81177	5.78725	4.78054	90	90	90	92.70	3.00
0.9	10.326	6.0013	4.7036	90	90	90	1.72	9.8171	5.79008	4.78293	90	90	90	98.28	2.25
1.0	10.3202	6.0020	4.6940	90	90	90	3.14	9.8100	5.78640	4.78018	90	90	90	96.86	3.78

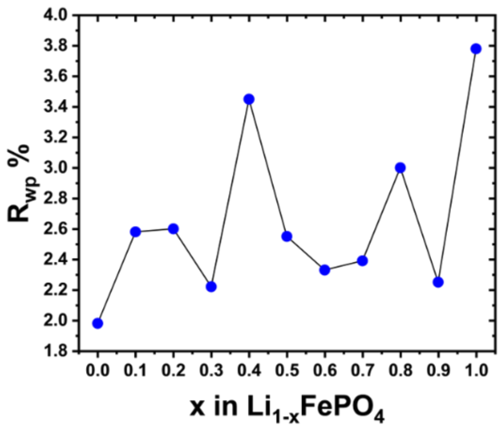

Figure S4: R_{wp} values obtained after Rietveld refinement as a function of lithium removal.
